# Transcorneal Electrical Stimulation Modulates Visual Pathway Function in Mice

**DOI:** 10.1002/jnr.70026

**Published:** 2025-02-11

**Authors:** Valerio Castoldi, Elena Rossi, Silvia Marenna, Giancarlo Comi, Letizia Leocani

**Affiliations:** ^1^ Experimental Neurophysiology Unit Institute of Experimental Neurology (INSPE) – IRCCS San Raffaele Scientific Institute Milan Italy; ^2^ Faculty of Medicine Università Vita‐Salute San Raffaele Milan Italy; ^3^ Department of Neurorehabilitation Sciences Casa di Cura Igea Milan Italy

**Keywords:** electroretinogram, neuromodulation, optic nerve, retina, visual evoked potential

## Abstract

Due to its ability to modulate neuronal activity, electrical stimulation of the eye may be a promising therapy for preserving or restoring vision. To investigate how electrical currents can influence visual function, Transcorneal Electrical Stimulation (TES) was tested on both female and male C57BL/6 mice to evaluate its neuromodulatory effect from the retina to the cerebral cortex through visual evoked potential (VEP) and electroretinogram (ERG) recording. VEP or ERG was acquired before (baseline), immediately (t0), after 5 min (t5), and 10 min (t10) of sham (i.e., no stimulation) or TES applied on the eye of anesthetized C57BL/6 mice. Notably, TES affected neuronal activity in the visual pathway since a significant increase in VEP and ERG amplitude was detected and persisted 10 min after TES. The amplitude increase induced by TES could underlie an enhancement of neuronal excitability that may ameliorate retinal‐genicular‐cortical function in diseases involving the visual system.

## Graphical abstract

Non‐invasive Transcorneal Electrical Stimulation (TES) applied on anesthetized C57/BL6 mice was capable of modulating visual pathway functionality since a significant increase in visual evoked potential (VEP) and electroretinogram (ERG) amplitude was detected immediately (t0), 5 min (t5), and 10 min (t10) after stimulation.

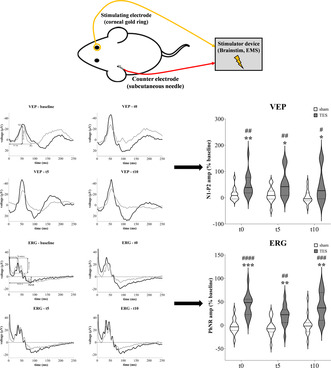

**Summary**

- Low‐intensity electrical currents delivered non‐invasively to the brain can change the activity of the central nervous system, and increasing or decreasing the excitability of neurons may play a key role in preserving and/or restoring the neuronal functionality in several neurological diseases.
- In this work, sinusoidal currents were administered to the eyes of naïve mice, and interestingly, an increase in electrical activity in both the retina and visual cortex was found after stimulation, which could play a key role in ameliorating visual pathway function in neurodegenerative conditions.

## 1 Introduction

Electrical stimulation (ES) of the eye has emerged as a promising technique to improve visual deficits in several ocular diseases, such as retinitis pigmentosa, age‐related macular degeneration, optic neuropathy, glaucoma, and retinal artery occlusion (Liu et al. 2021). This technique involves applying controlled electrical currents to various structures within the eye. In humans, several devices are used to apply ES, such as retinal prostheses (known as retinal implants or chips), but to avoid invasive procedures, transpalpebral, transorbital, and transcorneal (TES) approaches are preferred, which utilize contact lens electrodes or ring electrodes that do not touch the eye. Before going to the clinic, in animal experiments that study the benefits of electrical stimulation, corneal electrodes are the first choice because of their easy fastening procedure, particularly ensuring a simple and reliable method for TES. Taking advantage of the ease of access to the central nervous system through the visual pathway, applying painless electrical currents may elicit specific physiological responses in the retina, optic nerve, lateral geniculate nucleus, and visual cortex. In recent years, TES has quickly emerged as a non‐invasive neuromodulatory technique that can influence not only retinal and optic nerve activity but also brain connectivity and functionality (Agadagba et al. 2023). Several research works have shown the efficacy of TES on the visual pathway using rats, cats, and rabbits as preclinical models (Sehic et al. 2016). Among these studies, it has been reported that TES could exert a neuroprotective effect on the retina (Morimoto et al. 2005). The same group systematically changed the current intensity, frequency, pulse duration, waveform, and the number of TES sessions, demonstrating that repeated stimulation after optic nerve transection was the most effective in protecting the retinal ganglion cells (RGCs) from death (Morimoto et al. 2010).

The neuroprotective mechanism led by TES seems to be driven by the elicited expression of neurotrophic factors (Sato et al. 2008; Ni et al. 2009), together with a reduction of inflammatory response in the retina (Jassim et al. 2021). However, little is known about the possible influence of TES on the visual system electrophysiology. In particular, it would be of great interest to understand if TES could not only preserve the neuronal structure in the visual pathway but also its functional conduction from the retina to the visual cortex in vivo. For this purpose, visual evoked potential (VEP) and electroretinogram (ERG) represent reliable tools to measure visual pathway functionality. In neurophysiology, VEP quantifies the electrical signals produced in the visual cortex in response to a visual stimulus, being a marker of the functional integrity of optic nerves, projections to the visual cortex of the brain and occipital cortex (Creel 2019). Among the different parameters that are usually evaluated, VEP amplitude, which is the potential difference recorded by the electrode placed over the visual cortex after the visual stimulus delivery, quantifies the power of the electrical signal produced along the optic nerve. VEP amplitude can be considered a marker of axonal integrity along the visual pathway, and it is severely blunted in neurodegenerative conditions (Diem et al. 2003; Holder 2004; Castoldi et al. 2022). In particular, the peak‐to‐peak amplitude of the N1‐P2 wave complex seems to be related to axonal conduction, as well as cortical excitability (You et al. 2011). Interestingly, VEP amplitude can be influenced by electrical currents delivered with transcranial Direct Current Stimulation (tDCS) in a polarity‐dependent way (Cambiaghi et al. 2011), proving that electrical stimulation can modulate neuronal activity in vivo. Furthermore, TES led to an increase in VEP amplitude in an animal model of optic nerve crush, restoring the conduction impairments of the visual pathway at a very early stage from the damage and protecting retinal axons from ensuing degeneration (Miyake et al. 2007).

A further biomarker of visual function is represented by the electroretinogram (ERG), which measures the activity of the neurons in the retina when exposed to a brief flash of light (Perlman 2001). In light‐adapted conditions, the photopic negative response (PhNR) is the ERG wave generated by the RGCs after photic stimulation (Viswanathan et al. 1999; Machida 2012; Prencipe et al. 2020). The quantification of PhNR amplitude allows the evaluation of the RGC functionality, which can be affected in several diseases involving the retina (You et al. 2013; Brecelj 2014; Porciatti 2015). Even in the human retina, electrical stimulation of the eye has been shown to modulate RGC activity measured by ERG, leading to a decrease in amplitude obtained during both anodal and cathodal stimulations (Blum et al. 2020). In our study, we applied sinusoidal TES, which seems more effective than other waveforms (i.e., biphasic waves) in influencing retino‐cortical activity (Su et al. 2022). Specifically, we took advantage of the mouse visual system to check if TES could influence the neuronal activity measured by VEP and ERG before and after the current delivery. Up to now, the neuromodulatory effects of non‐invasive eye stimulation have not been deeply investigated. Therefore, our study aimed to elucidate the neurophysiological aspects of TES, which could have important implications for neuroprotection and neuronal connectivity restoration in human neurological disorders.

## 2 Methods and Materials

### 2.1 Experimental Subjects

The present study included 56 (*n* = 56) naïve C57/BL6 mice (28 males and 28 females between 8 and 22 weeks of age) housed under controlled temperature and on a 12 h light/dark cycle with chow pellets and tap water *ad libitum*.

### 2.2 Ethical Approval

The experimental procedures were performed according to the Animal Research: Reporting of In Vivo Experiments (ARRIVE) guidelines and were conducted in compliance with the Guide for the Care and Use of Laboratory Animals of the U.S. National Institutes of Health (NIH), the European Community guidelines (Directive 2010/63/EU), and the San Raffaele Institutional Animal Care and Use Committee (IACUC).

### 2.3 Experimental Design

Mice were divided into four groups of 14 mice each (7 males and 7 females) that underwent two different experimental protocols. In the first protocol, two groups were subjected either to baseline VEP (*n* = 14) or ERG (*n* = 14) recorded from the right eye; subsequently, 10 min of sham/real stimulation was applied to the right eye of each mouse. Then, VEP or ERG was acquired immediately (t0), 5 min (t5), and 10 min (t10) after stimulation. For the second protocol, two groups underwent baseline VEP (*n* = 14) or ERG (*n* = 14) from the left (*n* = 7) or right (*n* = 7) eye; afterward, 10 min of sham/real stimulation was delivered to the contralateral eye of each mouse. Therefore, VEP or ERG from the non‐stimulated eye was recorded immediately (t0), 5 min (t5), and 10 min (t10) after stimulation. In the two protocols, to avoid possible long‐lasting effects of both TES and anesthesia that could influence VEP or ERG parameters, sham stimulation was performed first, and then TES was applied on the same mice after seven days of washout. At the end of each experimental session, mice were put back in their home cage, where they could move and fully orient within the following 10–15 min.

### 2.4 Non‐Invasive Flash VEP and Photopic ERG Recording

Flash VEP and photopic ERG were performed as similarly published elsewhere (Marenna et al. 2020; Rossi et al. 2023). Briefly, mice were anesthetized with an intraperitoneal injection of 90 mg/kg ketamine (Ketavet—Intervet Productions s.r.l., Aprilia‐Latina, Italy) and 10 mg/kg xylazine (Rompun—Bayer s.p.a., Milan, IT). Pupils were dilated with 0.5% Tropicamide (Visumidriatic—Visufarma s.p.a., Roma, IT), and 2% Hydroxypropylmethylcellulose (GEL 4000—Bruschettini s.r.l., Genova, IT) was applied to avoid eye drying. Body temperature was maintained at 36.5°C ± 0.5°C during the experimental procedures with a homoeothermic blanket system with a rectal thermometer probe (Harvard Apparatus—Holliston MA, USA). VEP was recorded using a 6 mm Ø Ag/AgCl epidermal cup electrode (SEI EMG s.r.l.—Cittadella, IT) placed on the shaved scalp over V1 contralateral to the stimulated eye (1 mm anterior to the interaural line and 2.5 mm lateral to the midline) and a needle electrode in the nose for reference. The cup was fixed with an electro‐conductive adhesive paste (Elefix EEG paste—Nihon Kohden, Japan) and connected via flexible cables to an amplifier (SystemPlus Evolution—Micromed s.p.a., Mogliano Veneto, IT). Before the recording procedure, mice were placed in a dark room and adapted to darkness for 5 min. For each VEP recording session, three trains of 20 flash stimuli of 260 mJ intensity, 10 μs duration, and 1 Hz frequency were delivered with a flash photostimulator (Micromed s.p.a.—Mogliano Veneto, IT) placed at 15 cm from the stimulated eye.

Photopic ERG was recorded after 10 min of light adaptation using a gold ring electrode (LKC Technologies—Gaithersburg MD, USA) placed on the cornea and connected via flexible cables to the Micromed amplifier, with a needle electrode in the nose for reference. For each ERG recording session, three trains of 10 flash stimuli of 260 mJ intensity, 10 μs duration, and 0.5 Hz frequency were delivered with the flash photostimulator placed at 10 cm from the stimulated eye.

VEP and ERG were acquired at a sampling frequency of 4096 Hz, coded with 16 bits and bandpass‐filtered between 1–100 Hz for the analysis of N1 and P2 components, 0.3–300 Hz for the detection of PhNR, and 5–70 Hz for the measurement of the b‐wave. A notch filter (50 Hz) was used to remove external electrical noise.

### 2.5 Transcorneal Electrical Stimulation (TES)

TES was administrated to the mice still under the effect of anesthesia used to acquire baseline VEP/ERG. In particular, TES was applied after baseline recordings for 10 min using the gold ring electrode placed on the eye, with a needle counter‐electrode inserted in the back of the neck, as similarly described previously (Jassim et al. 2021). The hydroxypropylmethylcellulose was applied between the ring electrode and the cornea to protect the eye and secure the electrode. The stimulating and counter‐electrodes were connected via flexible cables to a BrainSTIM device (EMS s.r.l.—Bologna, IT) used to deliver the electric current, consisting of a symmetric biphasic sine wave (intensity: 100 μA; frequency: 20 Hz). For sham stimulation, the same setting was adopted with the BrainSTIM device switched off.

### 2.6 Electrophysiological Analysis

**FIGURE 1:**
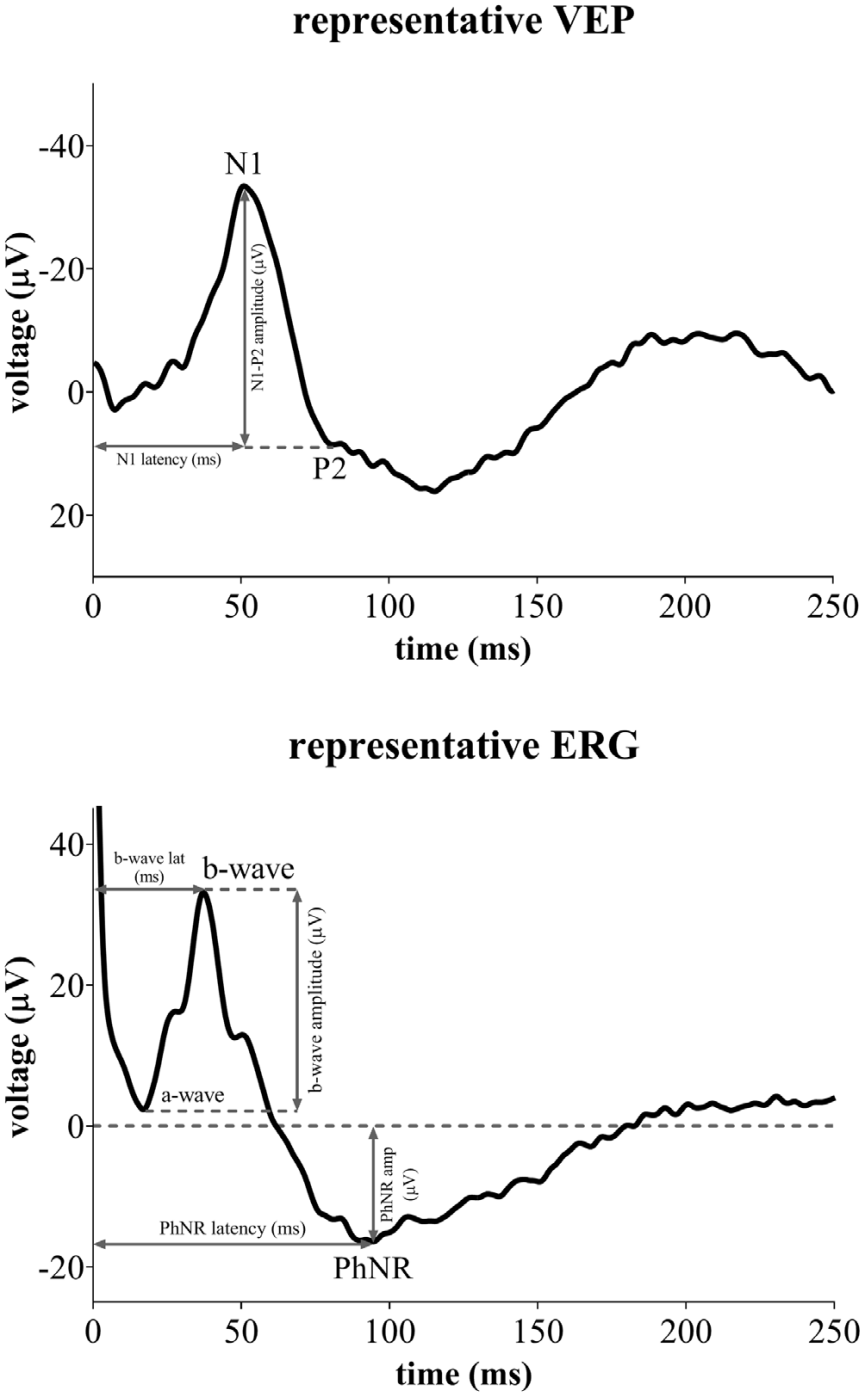
Representative VEP and ERG traces, with the N1, P2, PhNR, and b‐wave components highlighted.

VEP and ERG were measured offline with the Micromed SystemPlus Evolution software (Micromed s.p.a.—Mogliano Veneto, IT) used by a blinded operator unaware of the type of stimulation applied. For VEP, the peak‐to‐peak amplitude between the first negative (N1) and the second positive peak (P2) was quantified, together with N1 latency. Regarding ERG, the amplitude and latency of the Photopic Negative Response (PhNR: from baseline to the negative PhNR trough) and of the b‐wave (peak‐to‐peak between a‐ and b‐wave) were reported (Figure 1). Since significant differences were not found at baseline between sham and TES groups, all values were transformed as percentage changes from the baseline recording (before sham or real stimulation).

### 2.7 Statistical Analysis

Statistical analysis was performed using GraphPad Prism (version 9.3.1). To check possible gender effects, VEP and ERG were first compared using the mixed effects model, with “sex” (two levels: male and female), “time” (four levels: baseline, t0, t5, and t10), and “stimulation” (two levels: “sham” and “TES”) as main factors, adopting the Greenhouse–Geisser correction. VEP and ERG pooled from male and female mice were compared using the mixed effects model, with “time” (four levels: baseline, t0, t5, and t10) and “stimulation” (two levels: “sham” and “TES”) as main factors, applying the Greenhouse–Geisser correction. If “time” and/or “stimulation” effects were found, post hoc comparisons were performed using Dunnett's post hoc test for “time” factor and protected paired *t*‐tests for “stimulation” factor. Data were considered significant at *p* < 0.05.

## 3 Results

### 3.1 VEP and ERG Comparison Between Male and Female Mice

For all the VEP and ERG components analyzed, the effect of “sex” was not significant (ipsilateral N1‐P2 amplitude: *F*_1,12_ = 1.109, *p* = 0.192; contralateral N1‐P2 amplitude: *F*_1,12_ = 2.562, *p* = 0.135; ipsilateral N1 latency: *F*_1,12_ = 1.437, *p* = 0.254; contralateral N1 latency: *F*_1,12_ = 0.054, *p* = 0.821; ipsilateral PhNR amplitude: *F*_1,12_ = 0.497, *p* = 0.494; contralateral PhNR amplitude: *F*_1,12_ = 0.180, *p* = 0.679; ipsilateral PhNR latency: *F*_1,12_ = 0.877, *p* = 0.368; contralateral PhNR latency: *F*_1,12_ = 1.151, *p* = 0.304; ipsilateral b‐wave amplitude: *F*_1,12_ = 0.395, *p* = 0.542; contralateral b‐wave amplitude: *F*_1,12_ = 0.127, *p* = 0.728; ipsilateral b‐wave latency: *F*_1,12_ = 0.557; *p* = 0.470; contralateral b‐wave latency: *F*_1,12_ = 2.471; *p* = 0.142). Therefore, subsequent analyses were performed after pooling data from male and female mice.

### 3.2 VEP Modulation Induced by TES

**FIGURE 2:**
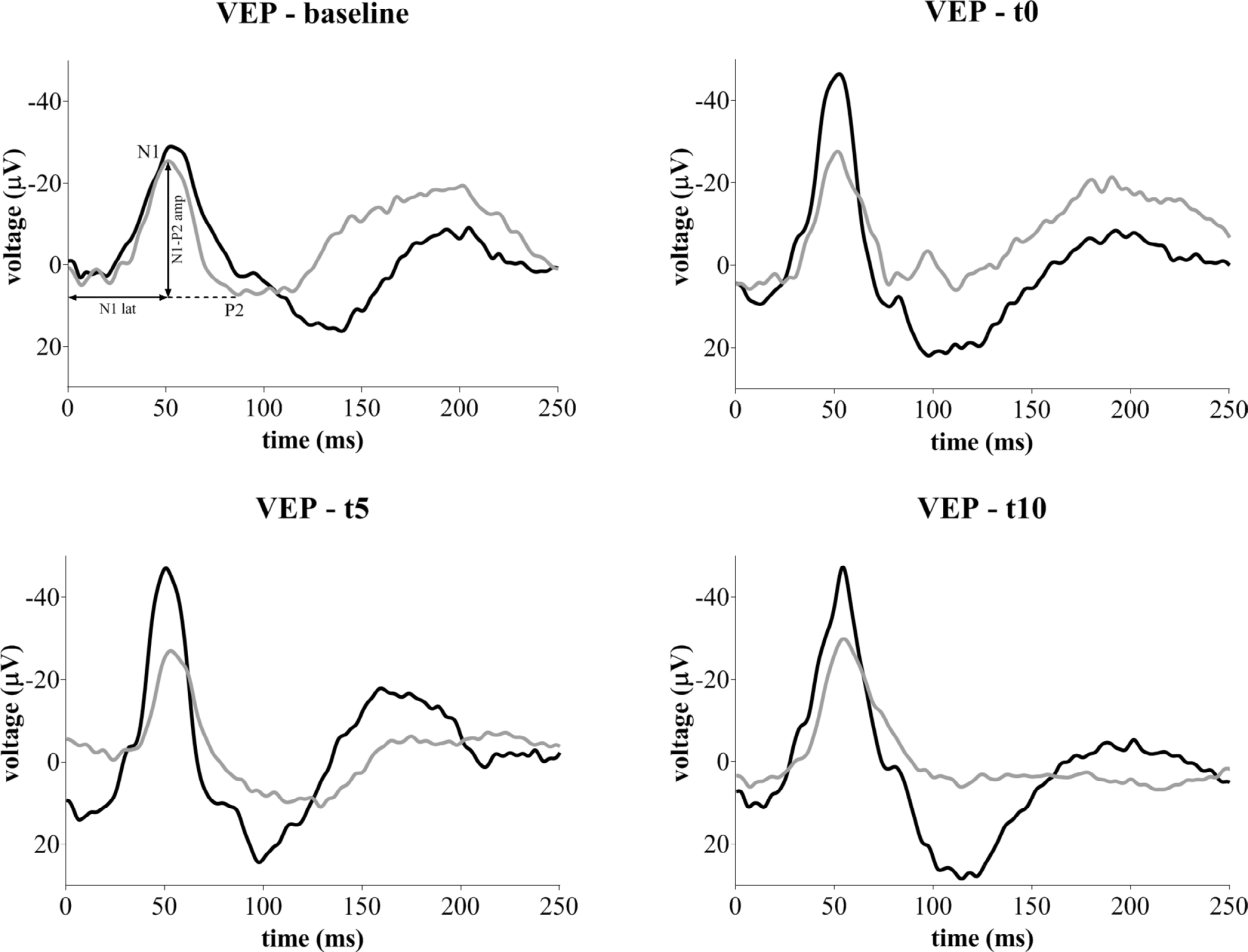
Representative VEP waveforms recorded from the ipsilateral eye of sham (gray line) and TES (black line) mice at baseline, t0, t5, and t10.

To find out the potential change of visual pathway functionality elicited by TES, baseline VEP (before stimulation) was compared with VEP recorded immediately (t0), 5 min (t5), and 10 min after sham or real stimulation of the ipsi and contralateral eyes. At visual examination, the amplitude of the N1‐P2 component recorded from the ipsilateral eye was higher in TES than in sham condition at t0, t5, and t10, while the N1 latency was similar (representative VEP traces recorded from single animals are reported in Figure 2). On the other hand, N1‐P2 amplitude and N1 latency acquired from the contralateral eye were visually comparable between sham and TES at every timepoint (Figure S1).

**FIGURE 3:**
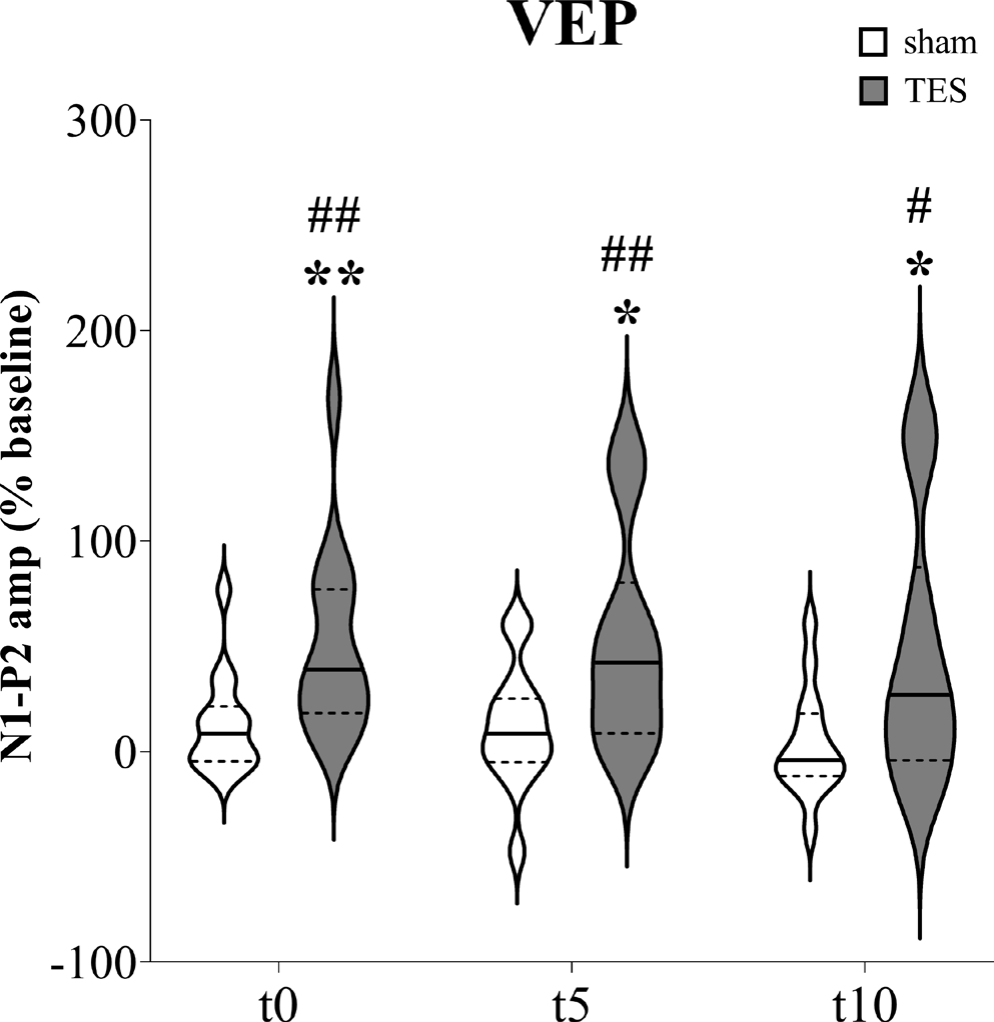
N1‐P2 amplitude percentage change from baseline measured immediately (t0), after 5 min (t5), and 10 min (t10) of sham stimulation or sinusoidal TES applied on the ipsilateral eye of naïve mice (*n* = 14). Hashes indicate significant differences after TES compared with baseline (^#^ *p* < 0.05; ^##^ *p* < 0.01); asterisks indicate significant differences between sham stimulation and TES at each timepoint (*p < 0.05; **p < 0.01). Mixed effects analysis with Dunnett's multiple comparisons for “time” factor (^#^) and paired *t*‐tests for “stimulation” factor (*). Data are expressed as violin plots with median (continuous line), first and third quartiles (dotted lines).

Considering the N1‐P2 amplitude quantification from the ipsilateral eyes (Figure 3), mixed effects analysis detected significant effects of “time” (*F*_2,60_ = 9.449; *p* < 0.001), “stimulation” (*F*_1,26_ = 8.194; *p* = 0.008), and “time × stimulation” interaction (*F*_3,78_ = 4.245; *p* = 0.008). Post hoc analysis revealed a significant increase in N1‐P2 amplitude at t0 (+50.9%; *p* = 0.002), t5 (+52.4%; *p* = 0.005), and t10 (+45.5%; *p* = 0.042) after TES compared with baseline. Conversely, sham stimulation did not change significantly the N1‐P2 amplitude at t0 (+12.8%; *p* = 0.149), t5 (+11.4%; *p* = 0.341), and t10 (+3.5%; *p* = 0.918). Protected paired *t*‐tests highlighted significant increases in N1‐P2 amplitude after TES compared with sham stimulation at t0 (+38.1%; *p* = 0.006), t5 (+41.0%; *p* = 0.027), and t10 (+42.0%; *p* = 0.025).

On the other hand, no significant effects of “time” (*F*_3,76_ = 1.307; *p* = 0.278), “stimulation” (*F*_1,26_ = 0.099; *p* = 0.755), and “time × stimulation” interaction (*F*_3,78_ = 0.178; *p* = 0.911) were reported for the N1‐P2 amplitude measured from contralateral eyes subjected to sham and real stimulation (Figure S2).

**FIGURE 4:**
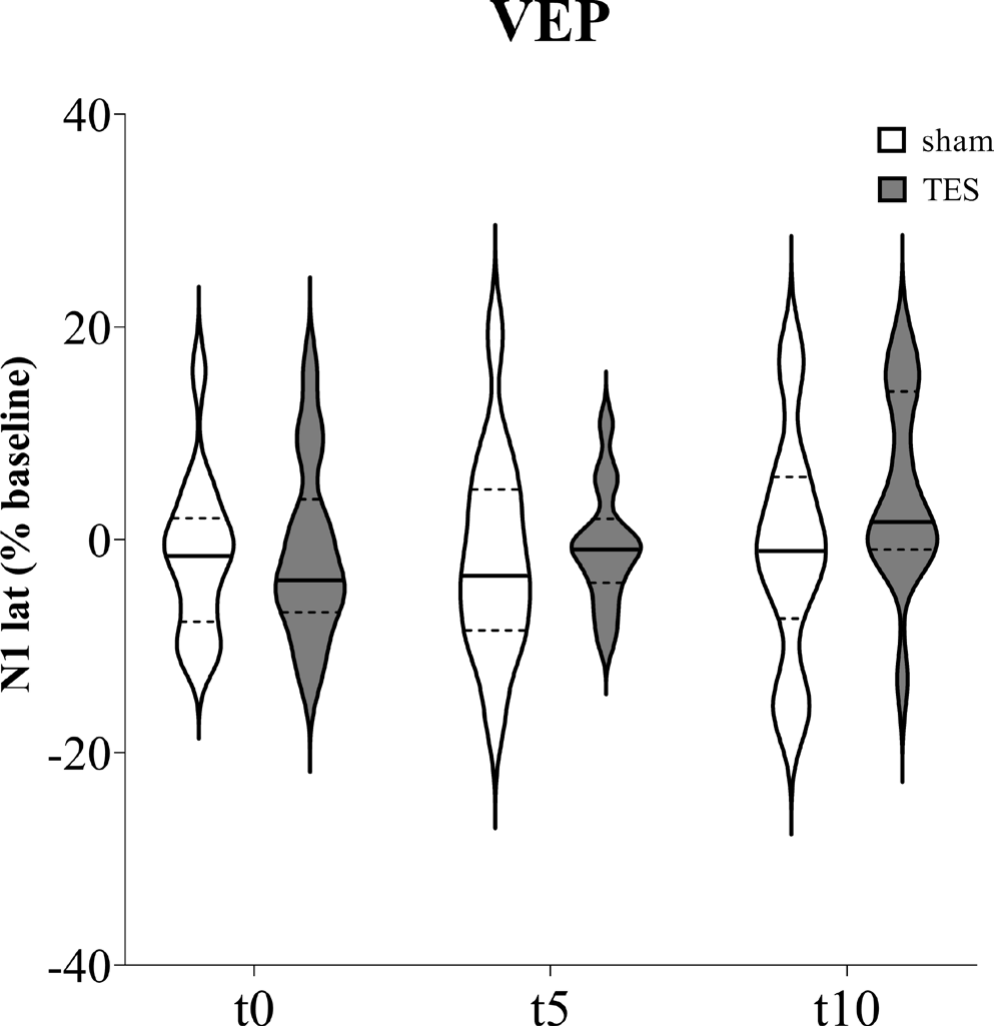
N1 latency percentage change from baseline measured immediately (t0), after 5 min (t5), and 10 min (t10) of sham stimulation or sinusoidal TES applied on the ipsilateral eye of naïve mice (*n* = 14). Data are expressed as violin plots with median (continuous line), first and third quartiles (dotted lines).

Concerning N1 latency recorded from ipsilateral eyes (Figure 4), mixed effects analysis did not find significant effects of “time” (*F*_3,69_ = 1.727; *p* = 0.175), “stimulation” (*F*_1,26_ = 0.654; *p* = 0.426), and “time × stimulation” interaction (*F*_3,78_ = 1.204; *p* = 0.314).

Even for N1 latency acquired from contralateral eyes (Figure S3), no significant effects of “time” (*F*_2,63_ = 1.747; *p* = 0.176), “stimulation” (*F*_1,26_ = 0.650; *p* = 0.427), and “time × stimulation” interaction (*F*_3,78_ = 1.025; *p* = 0.386) were observed.

### 3.3 ERG Modulation Induced by TES

**FIGURE 5:**
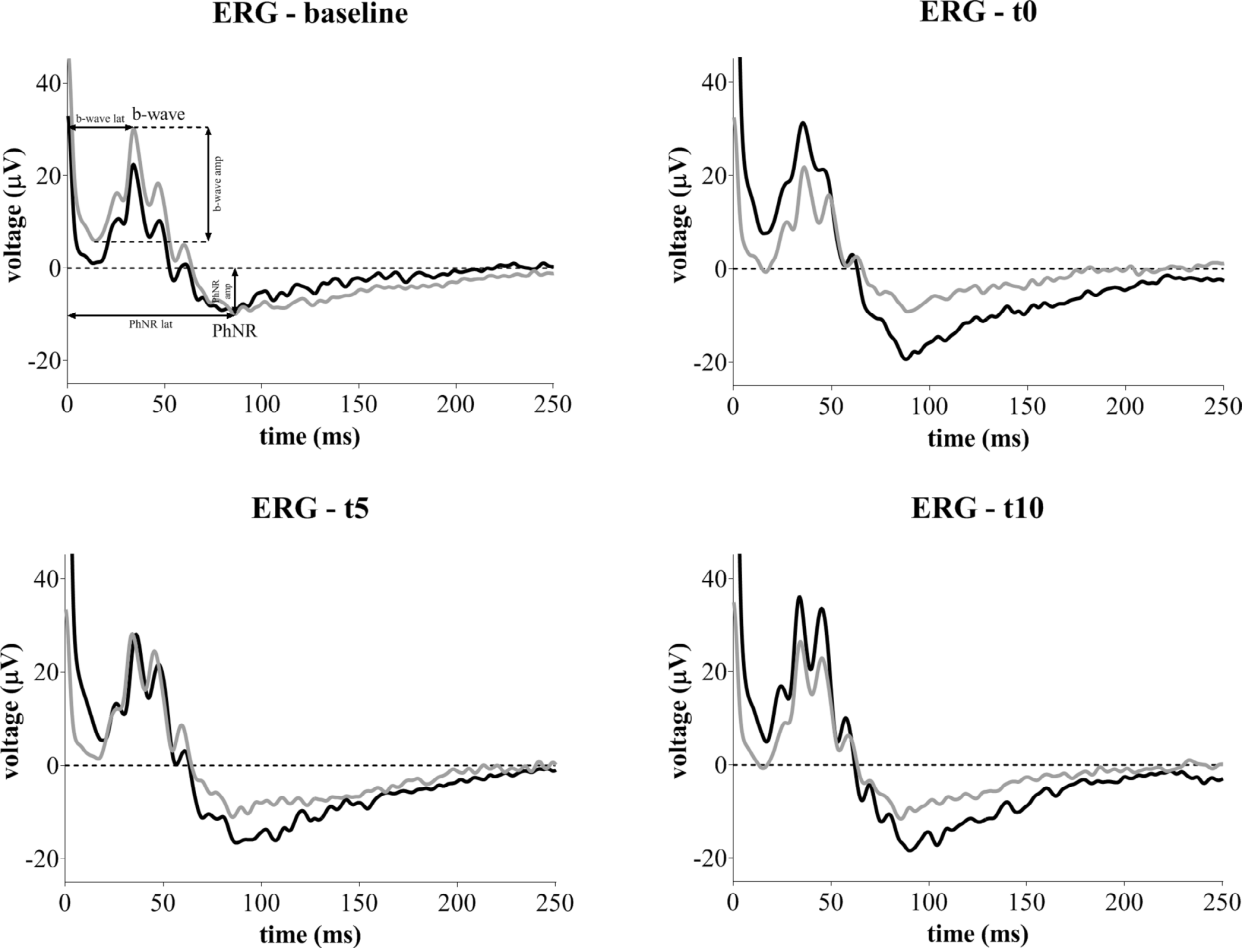
Representative ERG waveforms recorded from the ipsilateral eye of sham (gray line) and TES (black line) mice at baseline, t0, t5, and t10.

Aiming at discovering if TES could influence retinal function, baseline ERG (before stimulation) was compared with ERG recorded immediately (t0), 5 min (t5), and 10 min after sham or real stimulation of the ipsi and contralateral eyes. At visual inspection, the amplitude of the PhNR component acquired from the ipsilateral eye was higher in TES than in sham condition at t0, t5, and t10, whereas the PhNR latency was comparable. At some timepoints, an increase in b‐wave amplitude measured from the ipsilateral eye was detectable, even if it was quite variable over time, while b‐wave latency appeared stable (representative ERG traces recorded from single animals are shown in Figure 5). In contrast, the amplitude and latency of PhNR and b‐wave recorded from the contralateral eye were visually overlapping between sham and TES at every timepoint (Figure S4).

**FIGURE 6:**
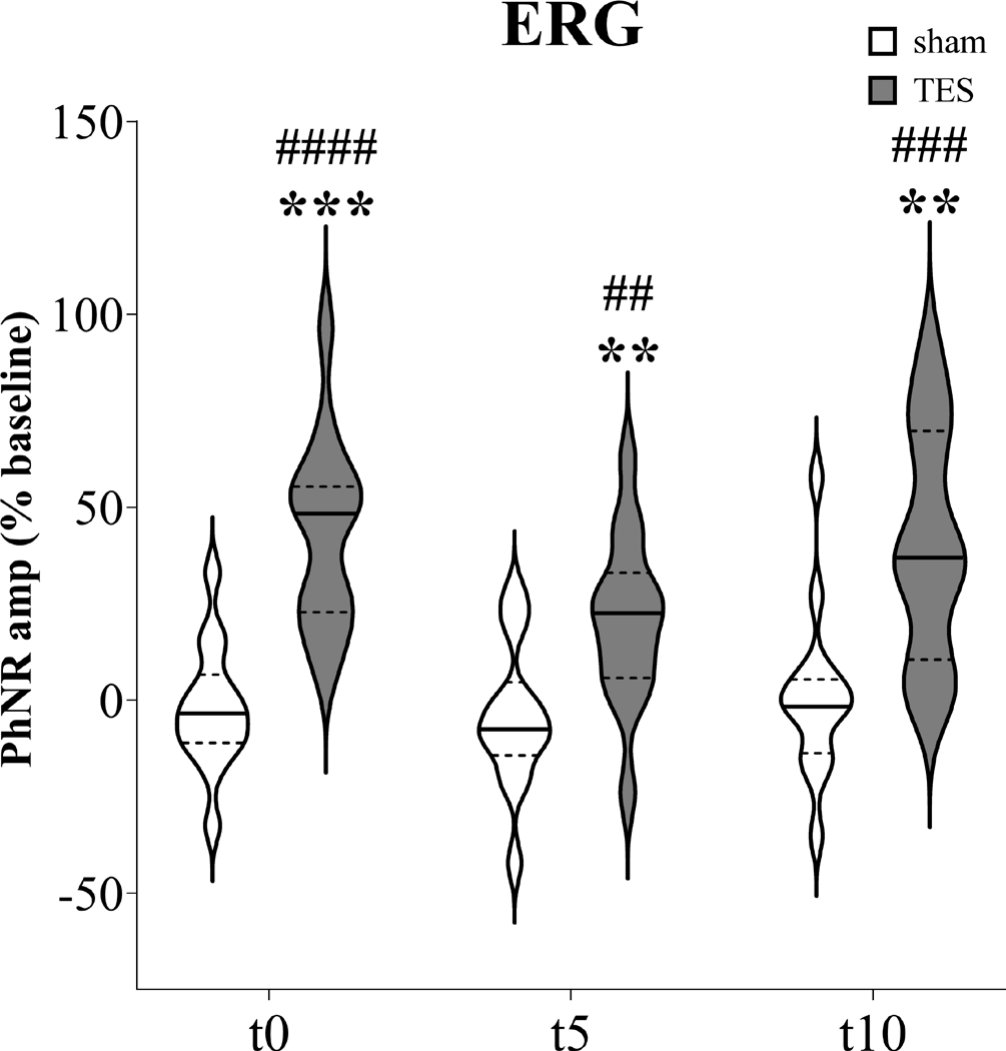
PhNR amplitude percentage change from baseline recorded immediately (t0), after 5 min (t5), and 10 min (t10) of sham stimulation or TES applied on the ipsilateral eye of naïve mice (*n* = 14). Hashes indicate significant differences after TES compared with baseline (^##^ *p* < 0.01; ^###^ *p* < 0.001; ^####^ *p* < 0.0001); asterisks indicate significant differences between sham stimulation and TES at each timepoint (**p < 0.01; ***p < 0.001). Mixed effects analysis with Dunnett's multiple comparisons for “time” factor (^#^) and paired *t*‐tests for “stimulation” factor (*). Data are expressed as violin plots with median (continuous line), first and third quartiles (dotted lines).

Regarding the PhNR amplitude quantification from the ipsilateral eyes (Figure 6), mixed effects analysis showed significant effects of “time” (*F*_2,65_ = 13.533, *p* < 0.0001), “stimulation” (*F*_1,26_ = 23.905; *p* < 0.0001), and “time × stimulation” interaction (*F*_3,78_ = 13.506, *p* < 0.0001). Post hoc analysis highlighted a significant increase in PhNR amplitude at t0 (+43.2%; *p* < 0.0001), t5 (+20.9%; *p* = 0.008), and t10 (+40.0%; *p* < 0.001) after TES compared with baseline. On the other hand, sham stimulation did not significantly change the PhNR amplitude at t0 (−2.1%; *p* = 0.928), t5 (−4.6%; *p* = 0.692), and t10 (+0.9%; *p* = 0.997). Protected paired *t*‐tests revealed significant increases in PhNR amplitude after TES compared with sham stimulation at t0 (+45.3%; *p* < 0.001), t5 (+25.4%; *p* = 0.008), and t10 (+39.1%; *p* = 0.002).

On the contrary, no significant effects of “time” (*F*_2,56_ = 2.778; *p* = 0.066), “stimulation” (*F*_1,26_ = 0.002; *p* = 0.961), and “time × stimulation” interaction (*F*_3,78_ = 0.926; *p* = 0.432) were reported for the PhNR amplitude measured from contralateral eyes subjected to sham and real stimulation (Figure S5).

**FIGURE 7:**
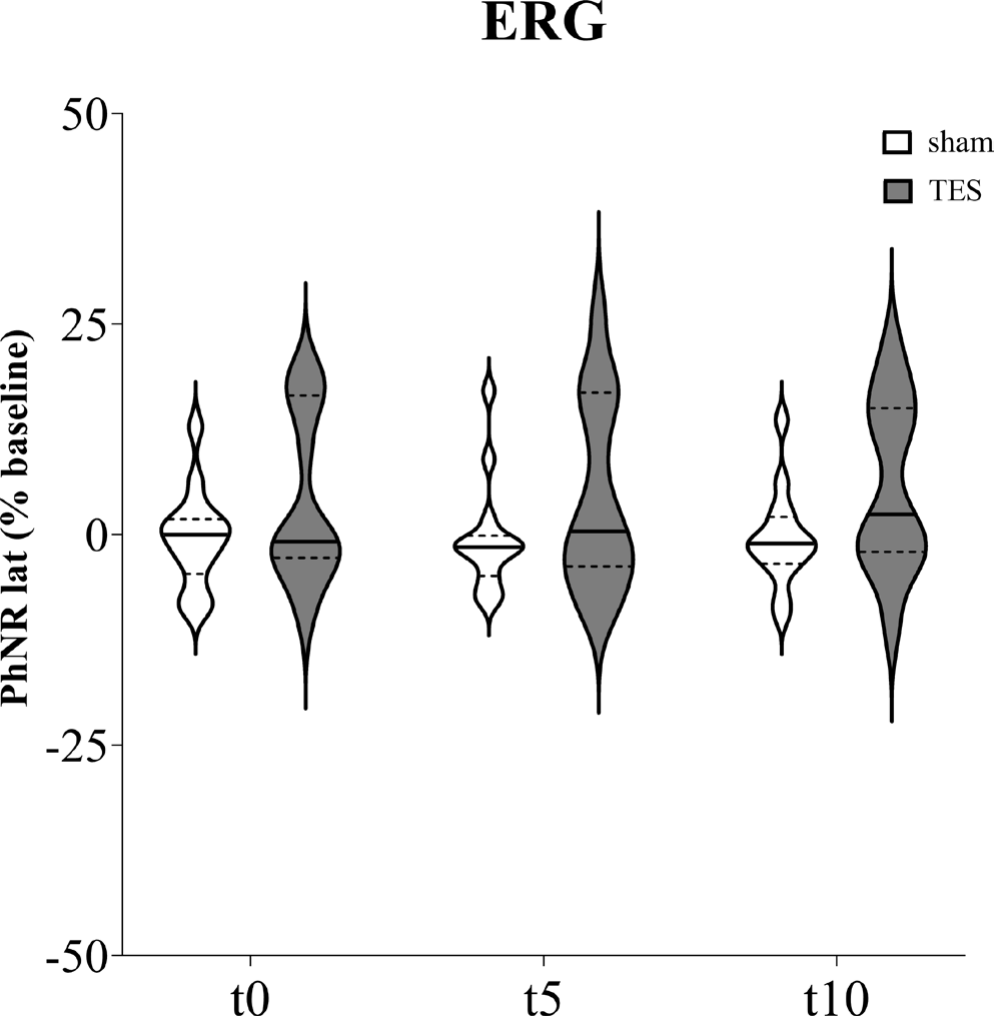
PhNR latency percentage change from baseline measured immediately (t0), after 5 min (t5), and 10 min (t10) of sham stimulation or sinusoidal TES applied on the ipsilateral eye of naïve mice (*n* = 14). Data are expressed as violin plots with median (continuous line), first and third quartiles (dotted lines).

With reference to PhNR latency recorded from ipsilateral eyes (Figure 7), mixed effects analysis did not detect significant effects of “time” (*F*_3,71_ = 0.885; *p* = 0.445), “stimulation” (*F*_1,26_ = 3.715; *p* = 0.065), and “time × stimulation” interaction (*F*_3,78_ = 1.366; *p* = 0.259).

Considering the PhNR latency from contralateral eyes (Figure S6), no significant effects of “time” (*F*_3,65_ = 2.180; *p* = 0.109), “stimulation” (*F*_1,26_ = 0.205; *p* = 0.655), and “time × stimulation” interaction (*F*_3,78_ = 2.013; *p* = 0.119) were found.

**FIGURE 8:**
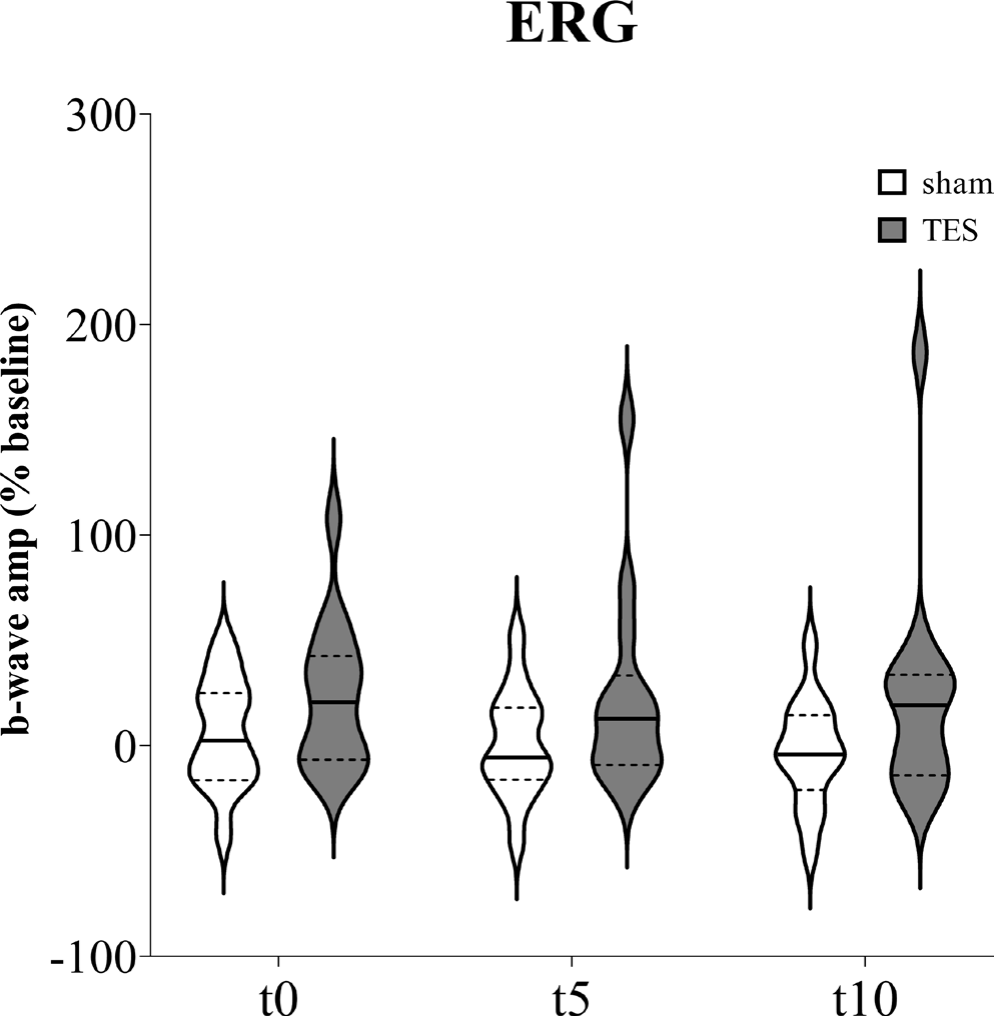
b‐wave amplitude percentage change from baseline measured immediately (t0), after 5 min (t5), and 10 min (t10) of sham stimulation or sinusoidal TES applied on the ipsilateral eye of naïve mice (*n* = 14). Data are expressed as violin plots with median (continuous line), first and third quartiles (dotted lines).

Taking into account the b‐wave amplitude measurement from ipsilateral eyes (Figure 8), mixed effects analysis did not highlight significant effects of “time” (*F*_2,43_ = 2.517, *p* = 0.101), “stimulation” (*F*_1,26_ = 2.750; *p* = 0.109), and “time × stimulation” interaction (*F*_3,78_ = 1.995, *p* = 0.122).

In the same way, mixed effects analysis of b‐wave amplitude from contralateral eyes (Figure S7) did not present significant effects of “time” (*F*_2,55_ = 2.720; *p* = 0.072), “stimulation” (*F*_1,26_ = 0.148; *p* = 0.704), and “time × stimulation” interaction (*F*_3,78_ = 0.152; *p* = 0.928).

**FIGURE 9:**
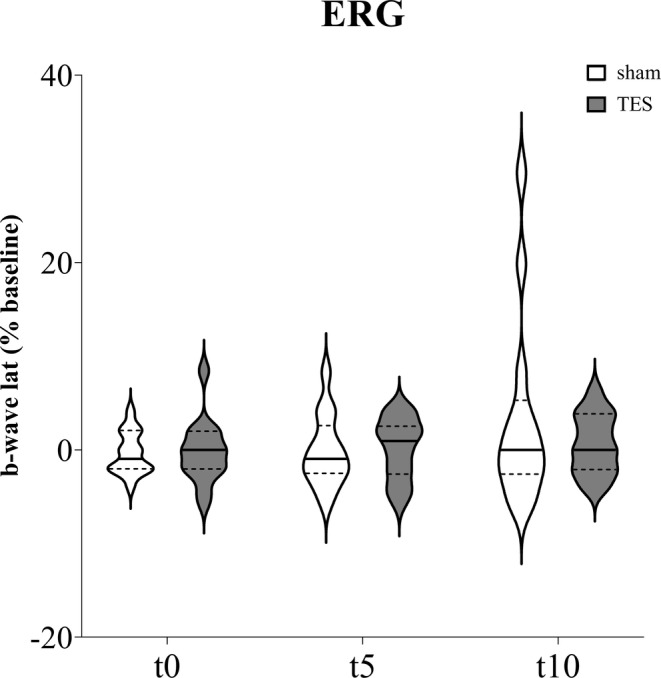
b‐wave latency percentage change from baseline measured immediately (t0), after 5 min (t5), and 10 min (t10) of sham stimulation or sinusoidal TES applied on the ipsilateral eye of naïve mice (*n* = 14). Data are expressed as violin plots with median (continuous line), first and third quartiles (dotted lines).

Concerning the b‐wave latency calculated from ipsilateral eyes (Figure 9), mixed effects analysis did not find significant effects of “time” (*F*_1,38_ = 1.984; *p* = 0.161), “stimulation” (*F*_1,26_ = 0.226; *p* = 0.638), and “time × stimulation” interaction (*F*_3,78_ = 0.958; *p* = 0.417).

Uniformly, mixed effects analysis of b‐wave latency from contralateral eyes (Figure S8) did not reveal significant effects of “time” (*F*_2,56_ = 2.932; *p* = 0.057), “stimulation” (*F*_1,26_ = 0.250; *p* = 0.621), and “time × stimulation” interaction (*F*_3,78_ = 0.602; *p* = 0.616).

## 4 Discussion

Non‐invasive electric or magnetic stimulation applied to target areas of the central nervous system is considered an intriguing strategy to modulate brain plasticity with important insights in the basic and translational research in the neuroscience field. To determine the capability of electrical stimulation to change neuronal activity, the evaluation of neural response before and after stimulation is essential and can be achieved through electrophysiological recording of the involved brain area using electroencephalography (EEG) and/or evoked potential (EP) acquisition. In our study, we focused on the visual system as an appropriate platform to test specific neuromodulation protocols due to its ease of access and the possibility of recording both retinal and cortical responses after the delivery of visual stimuli. In particular, we performed visual evoked potential (VEP) and electroretinogram (ERG) recordings to quantify the possible changes of visual pathway activity induced by non‐invasive electrical stimulation applied to the eye, named Transcorneal Electrical Stimulation (TES). Electrical stimulation delivered transcranially to naïve mice had already been capable of modulating the visual cortex activity (Cambiaghi et al. 2011), with anodal and cathodal currents that were able to increase and decrease the VEP amplitude, respectively. However, this amplitude change was detected immediately after stimulation and did not last long over time, returning to baseline levels between 5 and 10 min before the current delivery. Furthermore, the application of transcranial currents needs epicranial electrodes implanted through invasive surgery procedures, with the risk of accidental detachments and causing animal stress and suffering. For the present experiment, we followed a similar protocol adapted from Cambiaghi and co‐workers to check if TES could modulate the neuronal activity in both the ipsi and contralateral visual pathway, possibly for a more extended time. In addition to VEP recording, we performed ERG to specifically evaluate the TES effects on the retinal ganglion cells (RGCs) that project their axons to constitute the optic nerve. From a clinical point of view, inducing an amplitude increase in the ERG and VEP and therefore an increase of neuronal activity in the visual pathway would be useful for different purposes, such as eliciting neuroprotection (Morimoto et al. 2002; Miyake et al. 2007; Henrich‐Noack et al. 2017) and remyelination (Demerens et al. 1996; Gibson et al. 2014; Gautier et al. 2015) together with a reduction of inflammation (Colombo et al. 2021; Jassim et al. 2021) in neuropathological conditions. Therefore, we tested a sinusoidal waveform to try to obtain an overall modulation of the visual pathway, since sinusoidal currents have been reported to effectively change neuronal excitability in previous experimental settings (Freeman et al. 2010; Wang et al. 2020; Su et al. 2022). Moreover, the murine visual system has been widely studied to clarify the mechanisms of brain plasticity (Coleman et al. 2010; Hooks and Chen 2020), also guaranteeing high reliability of electrophysiological outcomes (Castoldi et al. 2021). Interestingly, we found a significant amplitude increase in both VEP and ERG after TES delivery to the ipsilateral eye compared with sham stimulation. Regarding the N1‐P2 amplitude component of VEP, we obtained an average increase between 45.5% and 52.4% with respect to the baseline condition (before stimulation). This result is in line with previous experiments in which electrical stimulation of the optic nerve (Gaillet et al. 2020) or retina (Miyake et al. 2007) induced an enhancement of cortical evoked potentials. The change in VEP amplitude after TES may be due to an increased excitability of the retina led by the RGCs that originate from the optic nerve. Subsequently, the increased electrical activity of the retina could propagate through the optic nerve to the synapses projecting from the lateral geniculate nucleus to the primary visual cortex. In this possible scenario, sinusoidal TES may be capable of modulating the visual cortex activity starting from the retina and covering the entire visual pathway. However, in our experimental setting, we cannot demonstrate that TES would directly or indirectly increase the excitability of the visual pathway downstream of the retina (i.e., the lateral geniculate nucleus and/or the primary and secondary visual cortex) since the VEP reflects the functional state of the entire visual system and cannot discriminate between the different neuronal structures. On the other hand, TES was particularly effective in modulating directly the retinal function, since the PhNR component of the photopic ERG acquired from the ipsilateral eye presented a significant increase in amplitude between 20.9% and 43.2% compared with baseline. As for VEP, this ERG change persisted after 10 min from stimulation, indicating that RGC spiking activity was more pronounced and sustained over time. The ERG amplitude increase may suggest a direct modulatory effect of TES on retinal function and could underlie an enhancement of the electrical activity in the retina (Morimoto et al. 2014) together with an increased expression of neurotrophic factors (Sato et al. 2008; Tao et al. 2016). Future works should include the detection of neurotrophic factors such as brain‐derived neurotrophic factor (BDNF) and glial cell line‐derived neurotrophic factor (GDNF) in the visual pathway to better characterize the molecular mechanisms behind TES. In the same way, ERG amplitude stabilization and/or increase after repeated TES sessions has been reported in clinical trials with patients affected by retinitis pigmentosa (Schatz et al. 2017; Sinim Kahraman and Oner 2020; Dizdar Yigit et al. 2022) and in mouse models of retinal degeneration, together with eliciting photoreceptors survival and neural progenitors proliferation (Yu et al. 2020). In our experiment, a decrease in PhNR amplitude occurred 5 min after TES (t5), even though it was still significant compared to the baseline and sham conditions. A possible explanation for this drop could be due to the level of sedation, which is difficult to control in mice anesthetized intraperitoneally. Each animal metabolizes chemical anesthetics differently, influencing the neuronal excitability and consequently the amplitude of evoked potentials.

Moreover, we did not notice significant increases in VEP and ERG amplitudes after TES administration to contralateral eyes, suggesting that the elicited neuromodulation would be confined to the ipsilateral visual tract, probably excluding an effect of the stimulation on the whole brain, or at least on the contralateral visual cortex. This may occur because the optic nerve fibers of the mouse cross almost entirely at the level of the optic chiasm (97%; Dräger 1974); therefore, the modulatory effect of TES might reach only the region of the visual cortex where the optic nerve of the stimulated eye projects. The scenario would be different in a possible application to humans, in which about half of the nervous fibers cross within the chiasm (53%; Kupfer et al. 1967) and in this case, we could not exclude a less focal and more widespread effect of TES at the cortical level.

Beyond the modulatory effects in the visual pathway, TES is also considered a tool to change neuronal plasticity in the central nervous system. In our setting, we were able to modulate the activity of the retina and visual cortex by directly stimulating the eye, reinforcing the scientific evidence that controlled delivery of electrical currents to the visual pathway could boost functional connectivity modulation in both experimental animals (Sergeeva et al. 2012; Agadagba et al. 2022) and humans (Gall et al. 2016; Sabel et al. 2020).

Notably, TES did not affect VEP and ERG latency, suggesting that sinusoidal stimulation did not change the speed of conduction from the retina to the visual cortex, mirroring the effect of tDCS on the visual pathway (Cambiaghi et al. 2011). The b‐wave component of the ERG, which is considered a marker of the activity of retinal bipolar, horizontal, and Müller cells (Sieving et al. 1994; Sustar et al. 2018), showed a slight increase in amplitude after TES compared to sham stimulation, although not statistically significant. This trend could be due to the opposite modulatory effects of TES on different cell populations in the inner retina, leading to neuronal hyper‐ or hypoexcitability reflected in the simultaneous increase or decrease in b‐wave amplitude.

Alongside the neuromodulatory properties, it should be important to underline the safety aspects of TES, especially in translational approaches. We did not perform histological analyses to check if TES could cause tissue damage, representing a study limitation that would be of interest in future works, particularly when applying TES for repeated sessions. However, we could assume that TES should not be harmful to the underlying tissues since sinusoidal currents were effective in preserving retinal function without altering retinal histology in an animal model of retinitis pigmentosa (Rahmani et al. 2013).

Taken together, our findings suggest that TES is a simple, efficient, and non‐invasive technique able to modulate the visual pathway function, leading to an increase in VEP and ERG responses that could play a central role in maintaining and promoting neuronal activity and survival against neurodegenerative conditions. Possible applications of TES other than retinitis pigmentosa or optic nerve injury may be considered, such as optic neuritis due to demyelination associated with functional alterations occurring in the visual pathway of patients affected by multiple sclerosis (Leocani et al. 2018; Petzold et al. 2022) and its animal models (Castoldi et al. 2018; Marenna et al. 2022). Regarding our experimental method, we did not obtain a neuromodulatory effect of TES on the whole brain, which may have important implications for nervous system dysfunctions involving areas other than the visual pathway. Intriguingly, TES also seems to improve cognitive functions in animal models of dementia (Yu, Aquili, et al. 2022) and depression (Yu, Tse, et al. 2022); however, in these works, the electrical stimulation was applied continuously for several days; therefore, we could hypothesize that consecutive TES sessions over time may influence regions of the brain that are not part of the visual cortex. In light of this hypothesis, it would be interesting to apply TES repeatedly to animal models of neurological diseases, particularly through follow‐up studies, to test possible long‐term therapeutic benefits. Therefore, the delivery of controlled electrical currents from the eye to the brain could represent a promising neuromodulatory treatment for patients suffering from both ocular and neurological diseases, in association with traditional therapeutic approaches or as an alternative to pharmacological treatments that retrieve unsatisfactory results.

Beyond any doubt, more experimental and clinical research efforts are needed to unravel the mechanistic insights that underlie the TES technique, trying to characterize its action in the retina, optic nerve, and possibly the whole brain, aiming to exploit its beneficial potential against the critical disorders of the central nervous system.

## 5 Declaration of Transparency

The authors, reviewers and editors affirm that in accordance to the policies set by the *Journal of Neuroscience Research*, this manuscript presents an accurate and transparent account of the study being reported and that all critical details describing the methods and results are present.

## Author Contributions

**Valerio Castoldi:** design, methodology, formal analysis, investigation, writing – original draft, visualization. **Elena Rossi:** formal analysis, investigation. **Silvia Marenna:** methodology, investigation. **Giancarlo Comi:** supervision, manuscript revision for intellectual content. **Letizia Leocani:** conceptualization, supervision, data interpretation, manuscript revision for intellectual content.

## Conflicts of Interest

The authors declare no conflicts of interest.

### Peer Review

The peer review history for this article is available at https://www.webofscience.com/api/gateway/wos/peer‐review/10.1002/jnr.70026.

## Supporting information


Data S1.

Transparent Science Questionnaire for Authors

## Data Availability

The data that support the findings of this study are available from the corresponding author upon reasonable request.
