# Supplementary material for: Transcorneal Electrical Stimulation Modulates Visual Pathway Function in Mice
Source: J Neurosci Res. 2025 Feb 11;103(2):e70026. doi: 10.1002/jnr.70026 (PMC11811922; doi:10.1002/jnr.70026)
Supplement: Supplementary file 1 — Data S1. [file JNR-103-e70026-s001.pdf]

Supplementary Material

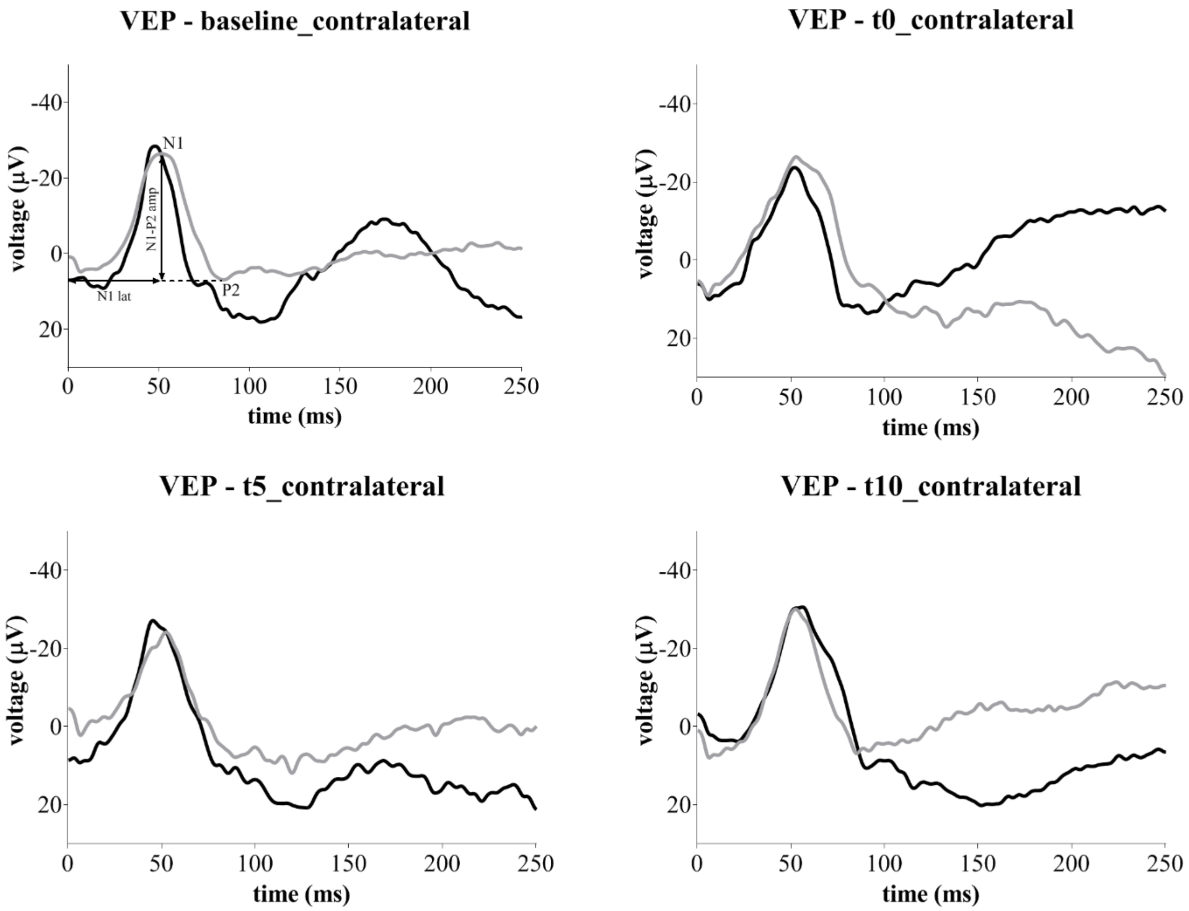

Fig S1. *Representative VEP waveforms recorded from the contralateral eye of sham (grey line) and TES (black line) mice at baseline, t0, t5 and t10.*

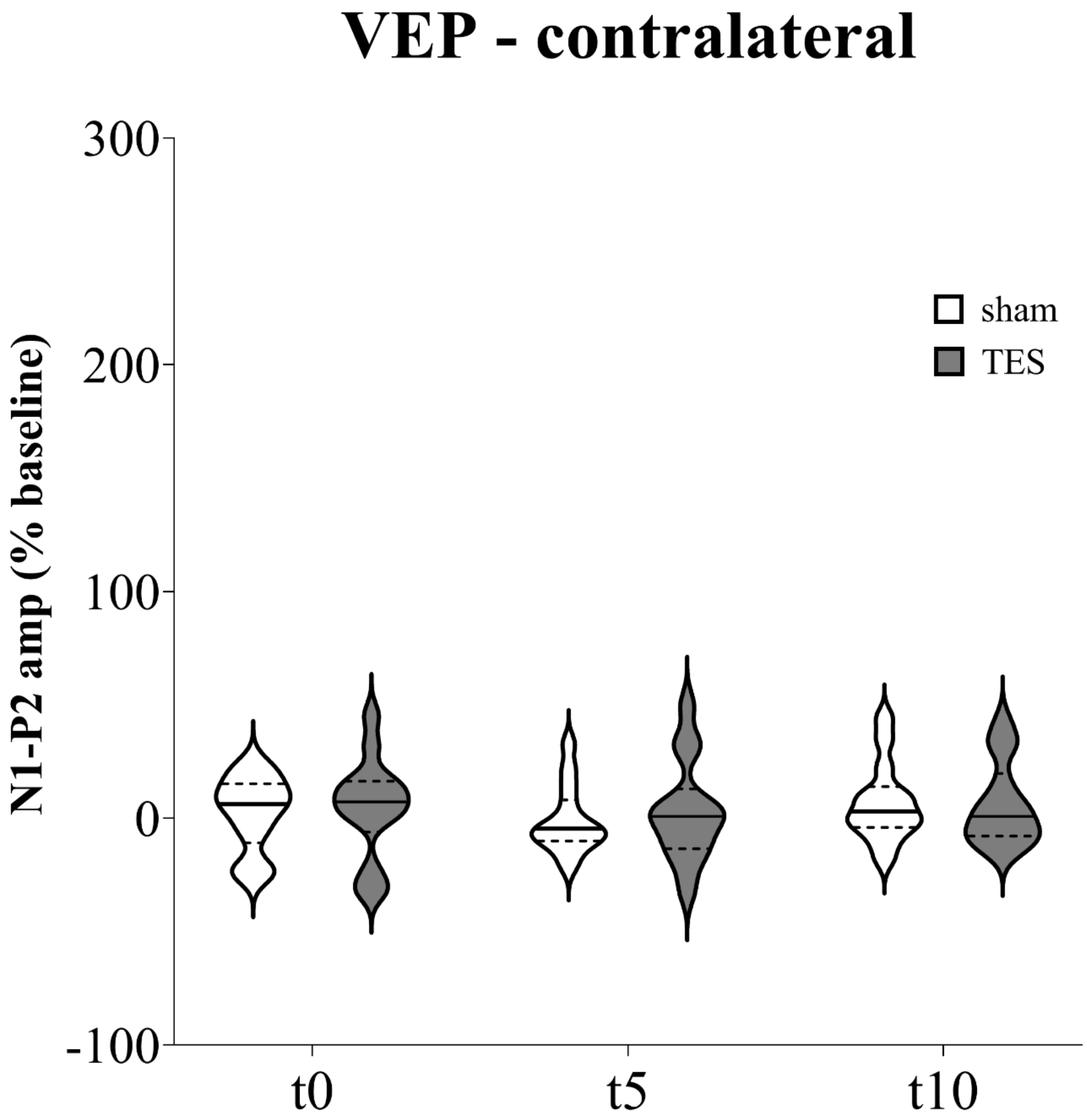

Fig S2: *N1-P2 amplitude percentage change from baseline recorded immediately (t0), after 5 (t5) and 10 minutes (t10) of sham stimulation or TES applied on the contralateral eye of naïve mice (n = 14). Data are expressed as violin plots with median (continuous line), 1st and 3rd quartiles (dotted lines).*

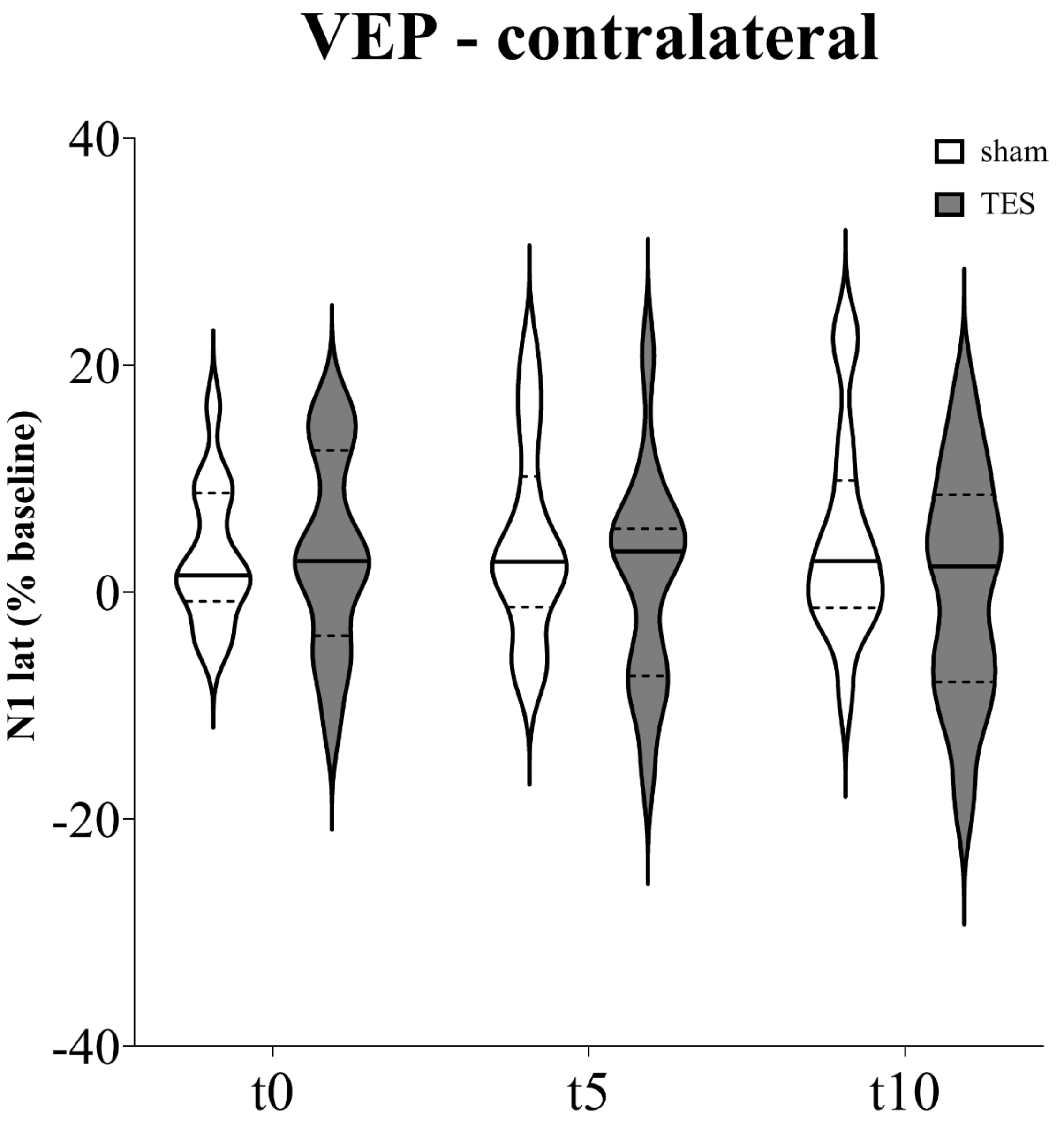

Fig S3: *N1 latency percentage change from baseline recorded immediately (t0), after 5 (t5) and 10 minutes (t10) of sham stimulation or TES applied on the contralateral eye of naïve mice (n = 14). Data are expressed as violin plots with median (continuous line), 1st and 3rd quartiles (dotted lines).*

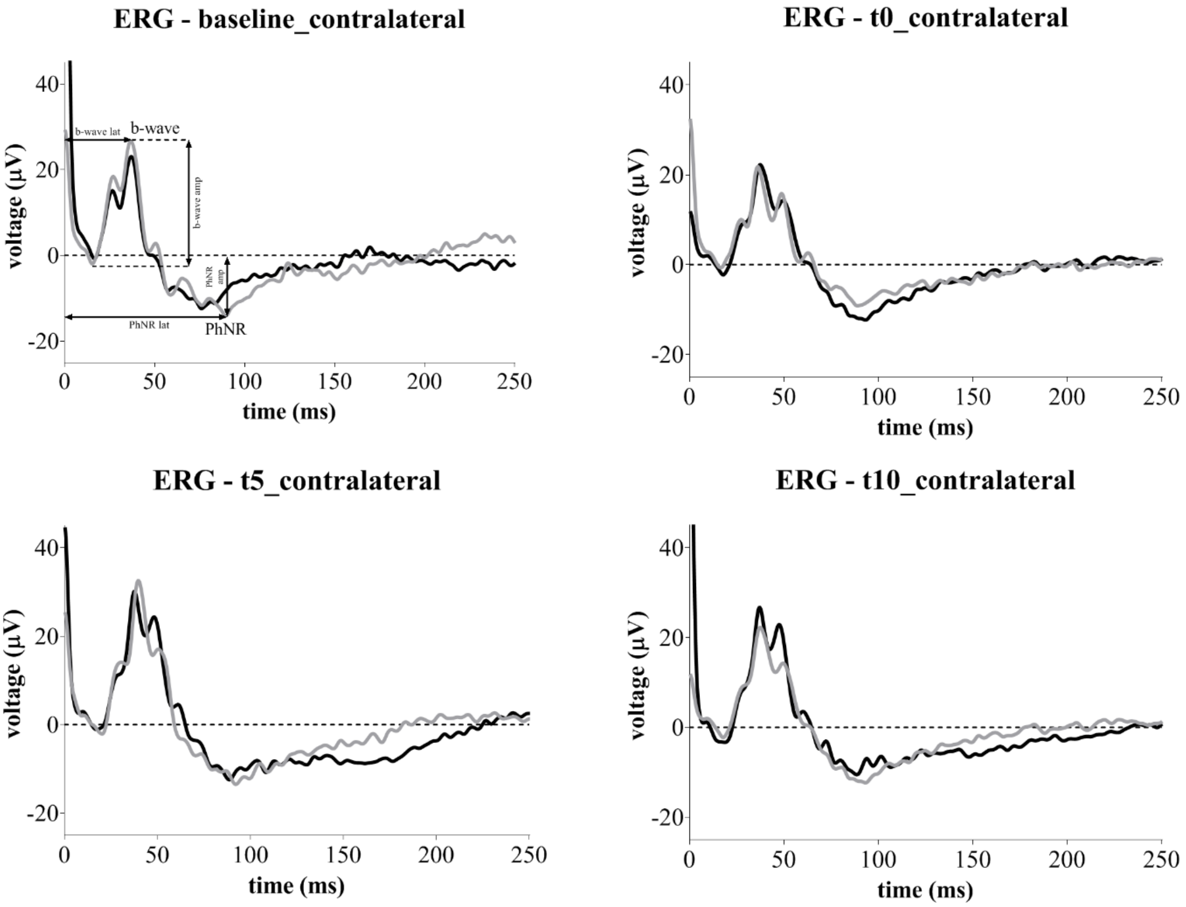

Fig S4. *Representative ERG waveforms recorded from the contralateral eye of sham (grey line) and TES (black line) mice at baseline, t0, t5 and t10.*

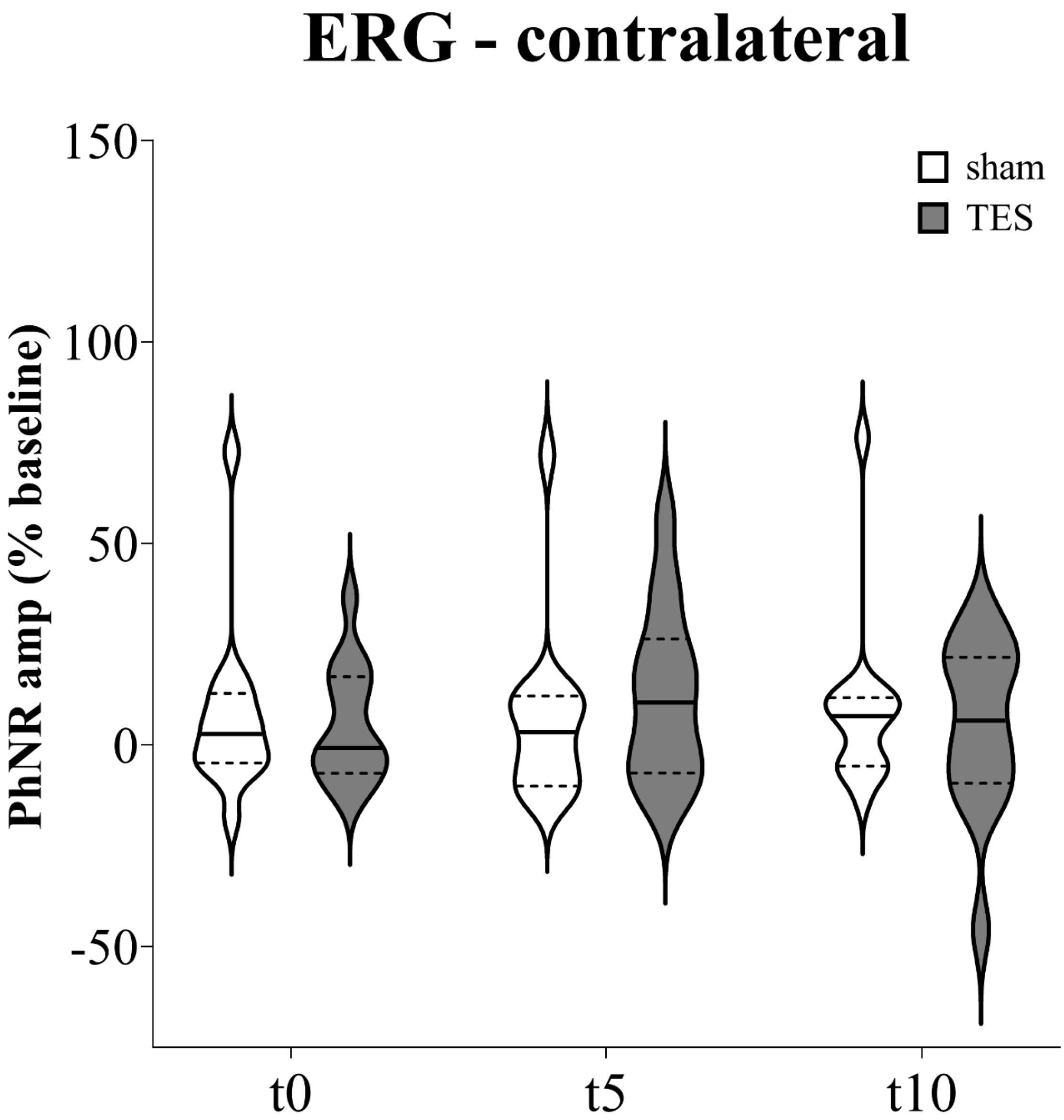

Fig S5: *PhNR amplitude percentage change from baseline recorded immediately (t0), after 5 (t5) and 10 minutes (t10) of sham stimulation or TES applied on the contralateral eye of naïve mice (n = 14). Data are expressed as violin plots with median (continuous line), 1st and 3rd quartiles (dotted lines).*

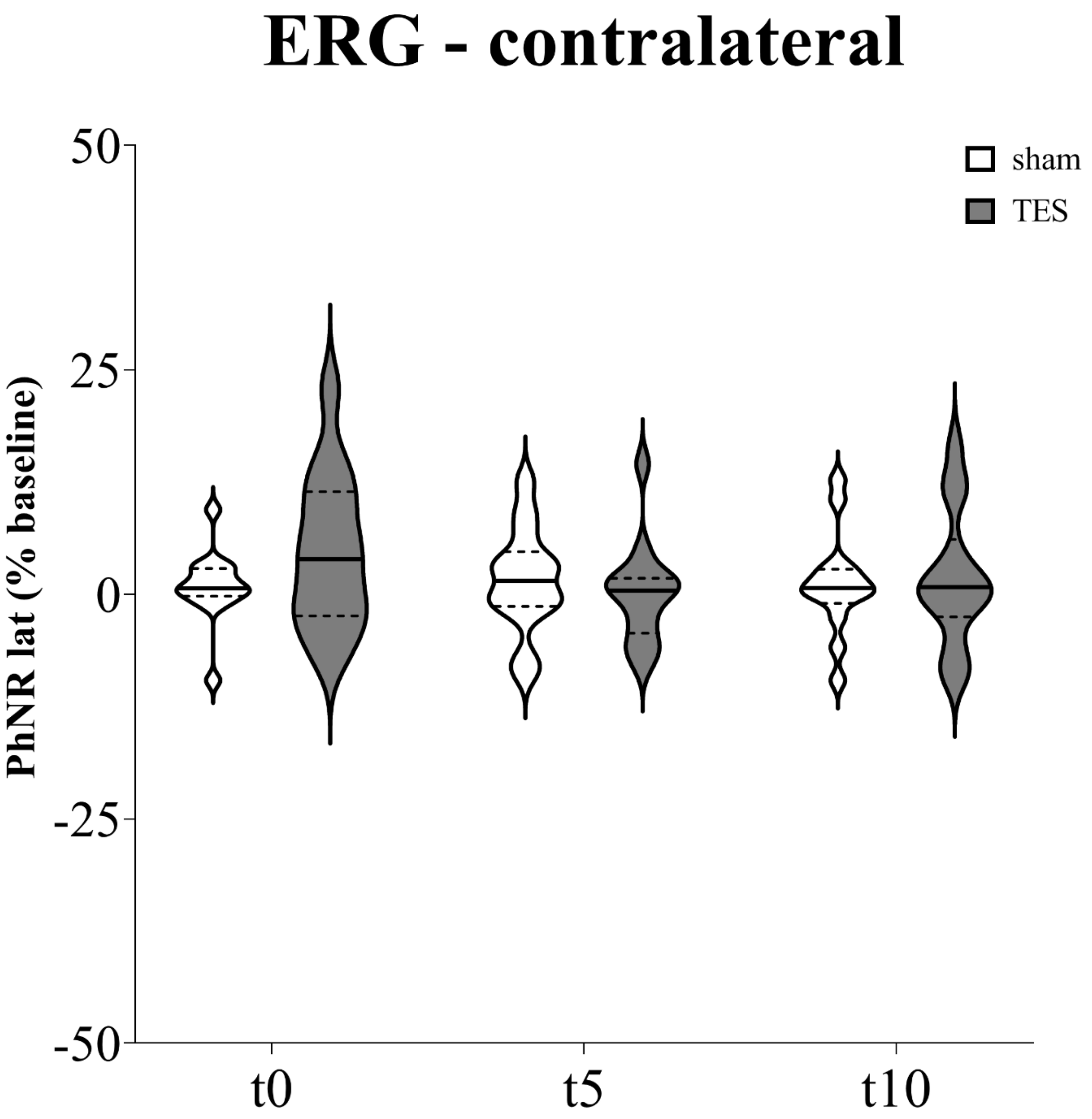

Fig S6: *PhNR latency percentage change from baseline recorded immediately (t0), after 5 (t5) and 10 minutes (t10) of sham stimulation or TES applied on the contralateral eye of naïve mice (n = 14). Data are expressed as violin plots with median (continuous line), 1st and 3rd quartiles (dotted lines).*

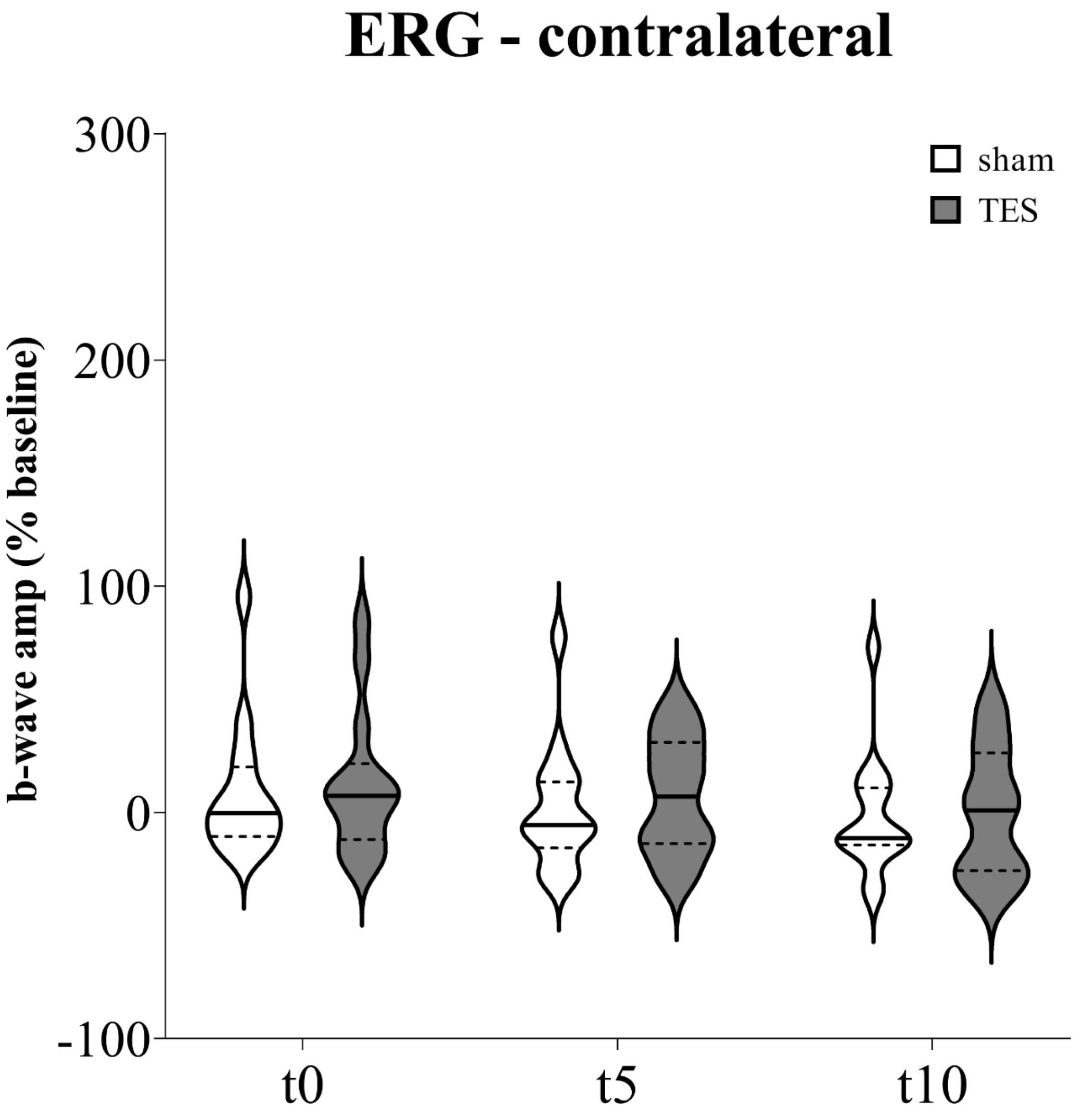

Fig S7: *b-wave amplitude percentage change from baseline recorded immediately (t0), after 5 (t5) and 10 minutes (t10) of sham stimulation or TES applied on the contralateral eye of naïve mice (n = 14). Data are expressed as violin plots with median (continuous line), 1st and 3rd quartiles (dotted lines).*

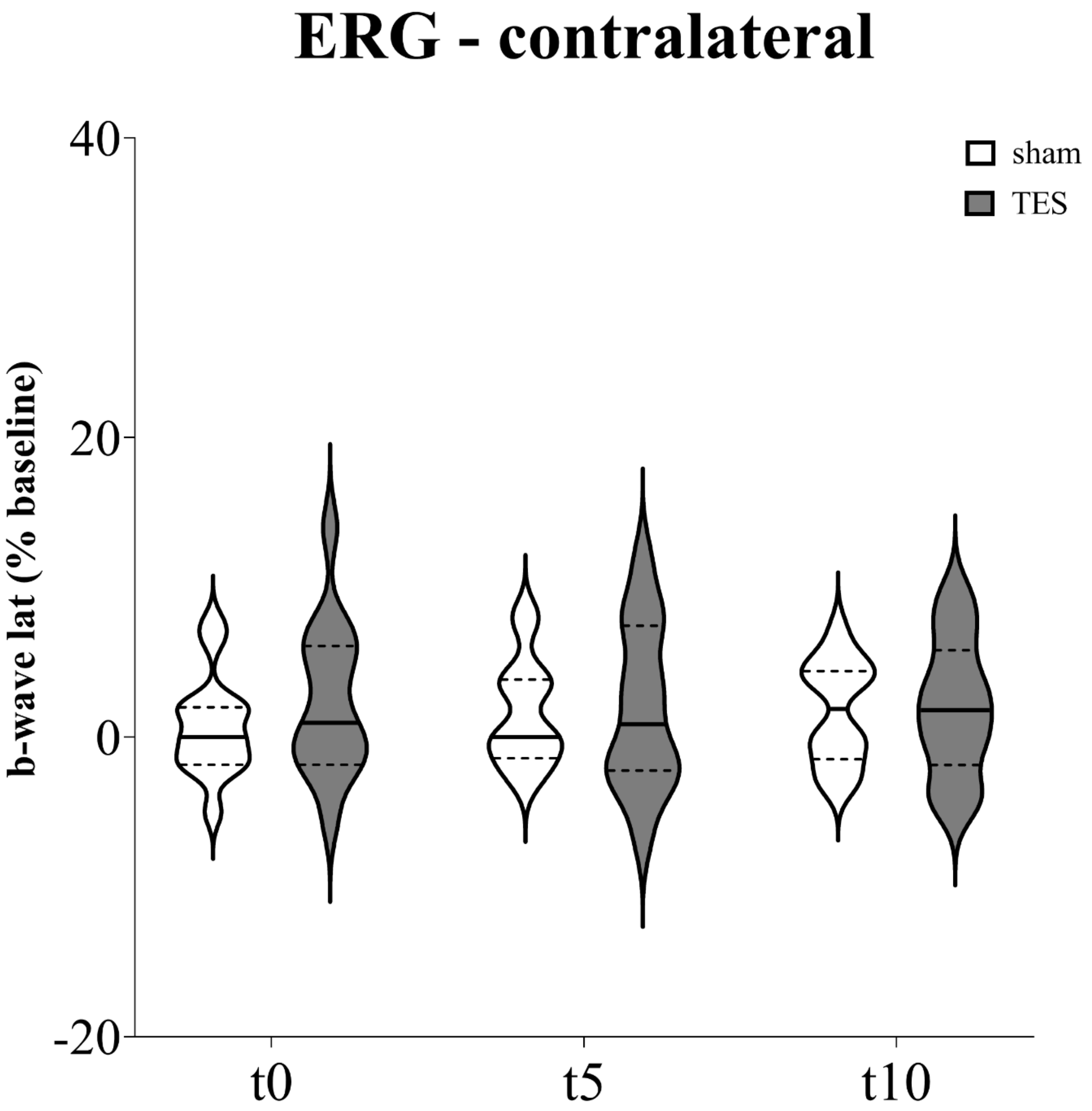

Fig S8: *b-wave latency percentage change from baseline recorded immediately (t0), after 5 (t5) and 10 minutes (t10) of sham stimulation or TES applied on the contralateral eye of naïve mice (n = 14). Data are expressed as violin plots with median (continuous line), 1st and 3rd quartiles (dotted lines).*
